# Percutaneous treatment of liver metastases

**DOI:** 10.1186/1470-7330-15-S1-O30

**Published:** 2015-10-02

**Authors:** Joseph P Erinjeri

**Affiliations:** 1 Interventional Radiology Service, Department of Radiology, Memorial Sloan Kettering Cancer, New York, New York, 10024 USA

## 

Early detection of metastatic disease within the liver by advanced diagnostic imaging has driven the rise of image-guided intervention for hepatic metastases. In this talk, we will explore the rationale, indications, technique and post procedure imaging findings of percutaneous treatments of liver metastases. Curative intent ablative therapies, such as radiofrequency ablation, microwave ablation, cryoablation, and irreversible electroporation will be discussed, including the common pitfalls of reading post ablative imaging studies. In addition, bridging and palliative therapies embolotherapies for liver dominant metastatic disease, such as hepatic artery embolization, transarterial chemoembolization, drug eluting beads, and yttrium-90 selective internal radiation therapy, will be reviewed.

